# A data set of a Norwegian energy community

**DOI:** 10.1016/j.dib.2021.107683

**Published:** 2021-12-06

**Authors:** Kjersti Berg, Markus Löschenbrand

**Affiliations:** a Dept. of Electric Power Engineering, NTNU, O.S. Bragstads plass 2E, Trondheim 7034, Norway; b Dept. of Energy Systems, SINTEF Energy Research, Sem Sælands vei 11, Trondheim 7034, Norway

**Keywords:** Energy community, Electric vehicles, Electrical power system, Household appliances, Local energy community, Photovoltaics, Scandinavia

## Abstract

This paper presents a data set designed to represent Norwegian energy communities. As such it includes household consumption data collected from smart meter measurements and divided into consumer groups, appliance consumption data collected from Norwegian households, electric vehicle data regarding charging patterns, simulated photovoltaic power generation data based on temperature and irradiance data sets and wholesale electricity prices.

All data sets are further filtered by season, weekday/weekend and time segment, and then fitted to either a normal, exponential or log-normal distribution. The reason for this specific segmentation is the intention to provide a suitable data set for case studies and experiments on energy communities that consider uncertainty, a main challenge to be overcome in the practical implementation of energy community projects. In addition to this filtered version, the previously unpublished raw data sets on household consumption and photovoltaic power generation are also provided.

## Specifications Table

**Table:** 

Subject	Electrical and Electronic Engineering
Specific subject area	The data describes electrical energy communities in the Norwegian power system. As such it contains consumption data from households, consumption data from household appliances, electric vehicle (EV) charging data, calculated power from photovoltaics (PVs) and wholesale electricity price.
Type of data	Table (.xlsx format)
How data were acquired	Household consumption: Smart meter measurements Appliance consumption: High-resolution measurements Electric vehicles: Secondary studies Photovoltaic generation: Agrometeorology Norway’s public repository Wholesale electricity price: Nordpool’s data repository
Data format	Raw Filtered
Parameters for data collection	At the core of the data collection was the intention for application in future simulation studies, thus an hourly resolution was mandated for the raw time series. In addition to this, and due to the filtering process (season, weekday/weekend, time segment), a considerable number of data points of over 5000 per time series was applied as a baseline.^1^
Description of data collection	Norwegian household consumption data from smart meters for 2015. Norwegian household appliance consumption data from the research project ElDek (2009–2012). Electric vehicle charging reports for Norwegian households from a separate study. Publicly available temperature and irradiance data for 2015–2021 from Agrometeorology Norway. Publicly available wholesale electricity prices for 2017–2021 from Nordpool.
Data source location	Country: Norway Photovoltaic generation - temperature and irradiance measurements: 15 weather stations in Norway (latitude, longitude): Alvdal (62.10944, 10.62687), Rakkestad (59.38824, 11.39042), Roverud (60.25378, 12.09144), Gjerpen (59.22684, 9.57805), Hjelmeland (59.22995, 6.14992), Lyngdal (58.13463, 7.04668), Mære (63.94244, 11.42527), Skjetlein (63.34038, 10.29737), Tingvoll (62.91341, 8.18623), Pasvik (69.45513, 30.04085), Tjøtta (65.82951, 12.42553), Holt (69.65381, 18.90946), Kvam (60.364277, 6.1748498), Njøs (61.179943, 6.862209), Ullensvang (60.31853, 6.65381). Wholesale electricity prices: Five Norwegian price areas (denoted commonly as NO1 - NO5). Primary data sources: Household consumption: Smart meter measurements from Trøndelag, Norway Appliance consumption: High-resolution measurements from Norway [1] Electrical vehicle charging: Measurements from Trondheim, Norway [2]
Data accessibility	Repository name: Mendeley Data Data identification number: 10.17632/y42z857vd5.3Direct URL to data: https://doi.org/10.17632/y42z857vd5.3Simulating photovoltaic generation: Repository name: GitHub Code identification number: 10.5281/zenodo.5647107Direct URL to code: https://github.com/kjeberg/FINE-PVgenFitting distributions: Repository name: GitHub Code identification number: 10.5281/zenodo.5723982Direct URL to code: https://github.com/Loeschenbrand/Sintef/blob/5ec05d0a5d9dd2fec9c7917af970b8f60a56e090/scripts/distributionfitting.py

1 Resulting in a number of $\frac{5000}{4}\cdot \frac{2}{7}\cdot \frac{4}{24}\approx 60$ data points for each weekend category.

## Value of the Data

• This data set consists of a composition of previously unpublished data sets, filtered data sets and simulated data. It allows for building stochastic case studies and simulations on energy communities within the Norwegian power grid.
• The target audience of the data set is researchers and decision makers in electric power systems that aim to implement simulations and experiments on realistic energy communities.
• The data set can be used in its current form to formulate case studies for various system topologies. In addition, it is possible to add information on the local grid, either from test systems or real networks. As such data sets are usually deterministic, the stochastic form of the here presented data complements this and allows for creating stochastic case studies of energy communities.
• Within Europe, a growing importance of decentralized energy community models can be observed. Aligned with this, in 2018 and 2019 respectively, the European Union has defined Renewable [3] and Citizen Energy Communities [4] as legal entities of such energy communities. This data set provides an opportunity to formulate heterogeneous test communities of varying sizes and varying characteristics such as number of EVs or available solar power generation capacity.

## 1 Data Description

**Fig. 1:**
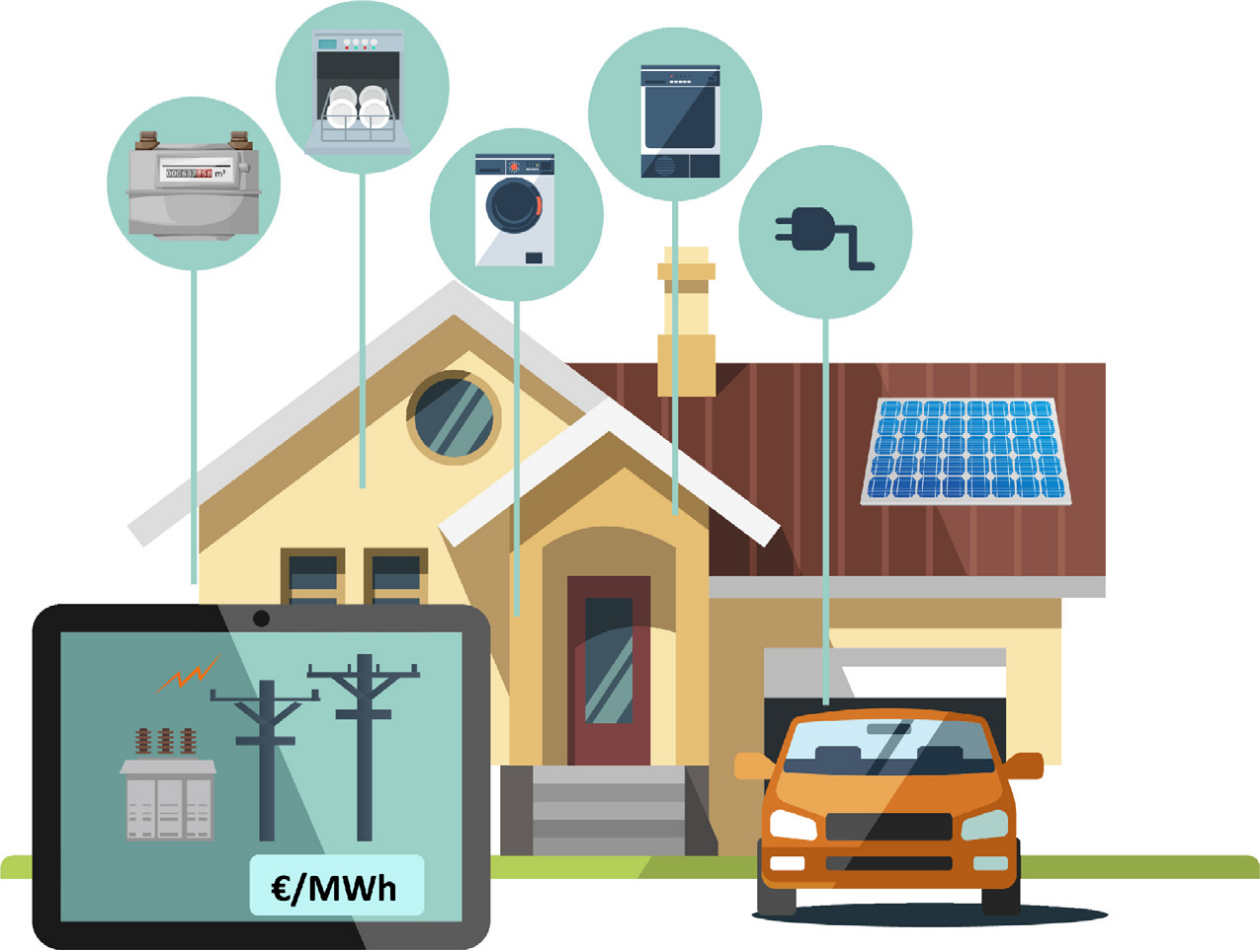
Exemplary overview.

A visual summary of all components in the data set is shown in Fig. 1.

In summary, the data set consists of five separate data sets as shown in Table 1. The data is presented as a single.xlsx-file consisting of seven individual sheets.

**Table 1:** Overview of data sets and sheets.

Data set	Sheet	Data in sheet
Household consumption^a^	*Households_raw*	Ratio of maximum load [ratio]
	*Households_filtered*	Filtering of groups A-D [ratio], fitted to a normal distribution.
Appliance consumption	*Appliances_filtered*	Ratio of maximum load for dishwasher, dryer and washing machine [ratio], fitted to an exponential distribution.
Electric vehicle charging	*EVcharging_filtered*	Filtered values of charging start probability [%] and charging duration [h], fitted to an exponential distribution.
Photovoltaic power generation	*PV_raw*	Power [Wh/h] for measuring locations in areas NO1-NO5
	*PV_filtered*	Filtered values [Wh/h] for areas NO1-NO5, fitted to a normal distribution.
Wholesale electricity price	*WholesalePrice_filtered*	Elspot price [Eur/MWh] for areas NO1-NO5, fitted to a log-normal distribution.

a Includes heating, which is in Norway most commonly conducted electrical [5].

All separate data sets contain filtered values. All filtered values, except the values for the appliances, are based on a three-dimensional filtering process. The segments of this process are shown in Table 2.

**Table 2:** Three-dimensional filtering process.

Dimension 1 - seasons	Dimension 2 - day of week	Dimension 3 - time of day
		00:00-04:00
Spring - March 21. to June 20.		04:00-08:00
Summer - June 21. to September 19.	Weekday (Monday to Friday)	08:00-12:00
Fall - September 20. to December 20.	Weekend (Saturday, Sunday)	12:00-16:00
Winter - December 21. to March 20.		16:00-20:00
		20:00-24:00

The filtering process follows four steps:

1. Separate the data corresponding to dimension 1 (seasons).
2. For each of the four segments created in Step 1, find the data corresponding to dimension 2 (day of week).
3. For each of the eight segments created in Step 2, find the data corresponding to dimension 3 (time of day).
4. For each of the 48 segments, fit the data to the given distribution.

The data for the appliances is not filtered by seasons, and therefore starts at step 2. Hence, only dimension 2 (day of week) and dimension 3 (time of day) are used to filter the appliance data, leading to 12 segments in total.

Table 3 shows a summary of the included data points, including starting and ending points of the utilized time series. Note that the data points for the simulated photovoltaic generation will be introduced further below in the following subsections. In addition to this, each individual component of the data set is also described in detail.

**Table 3:** Raw data set information.

Data set	Min. data points	Starting date	Ending date
Household consumption	4,944	01.01.2015 00:00	31.12.2015 23:00
Appliance - dishwasher	748	27.05.2010 00:00	02.12.2012 23:00
Appliance - dryer	420	01.06.2010 00:00	08.12.2012 23:00
Appliance - washing machine	676	07.05.2010 00:00	08.12.2012 23:00
EV charging	78	21.12.2018 10:00	31.01.2020 20:00
Wholesale electricity price	412	26.05.2017 10:00	04.02.2021 12:00

**Fig. 2:**
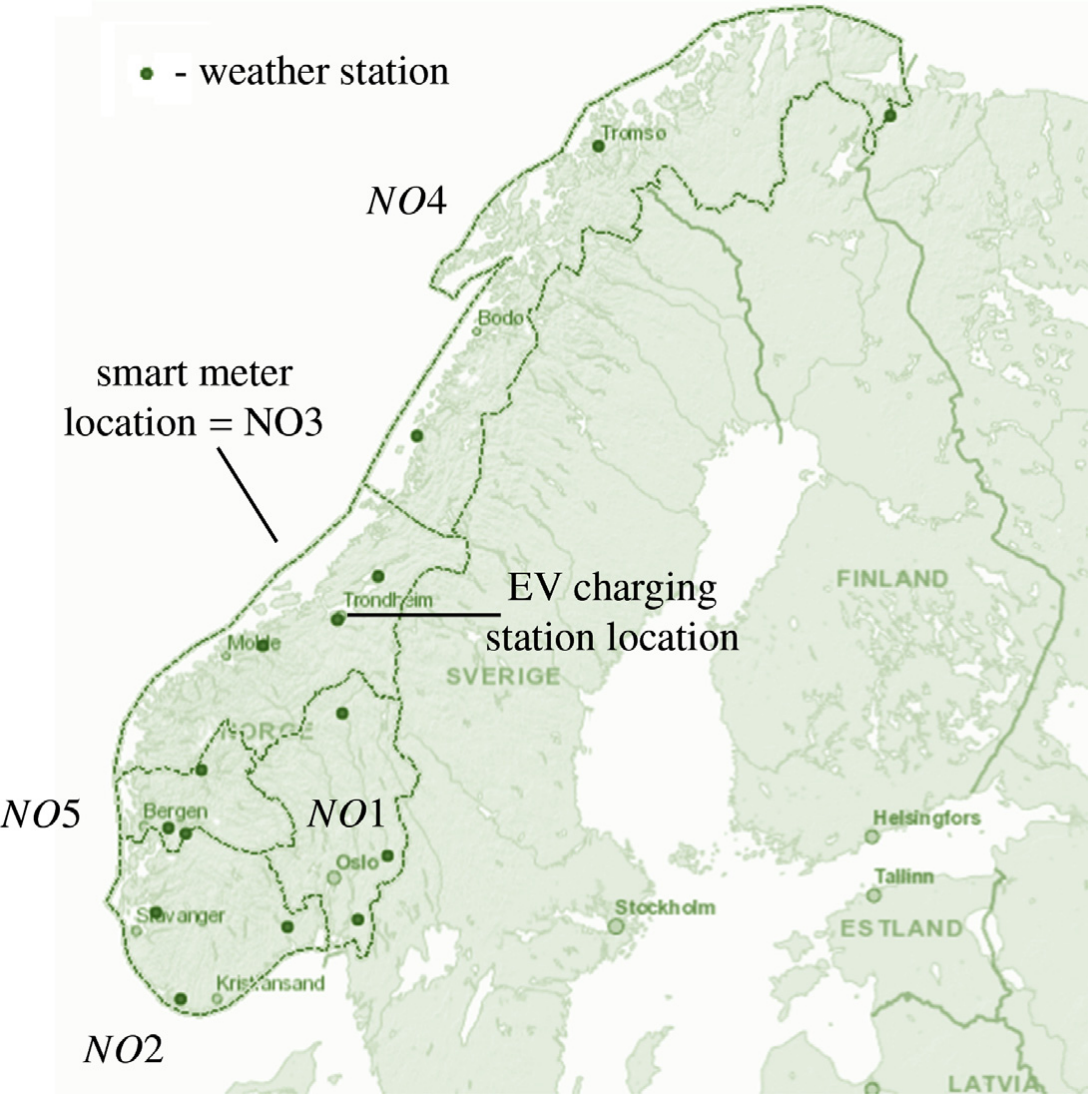
Data source location in the Norwegian power system.

Fig. 2 shows a geographical overview of the origin sites of the data set.

### 1.1 Household consumption

**Fig. 3:**
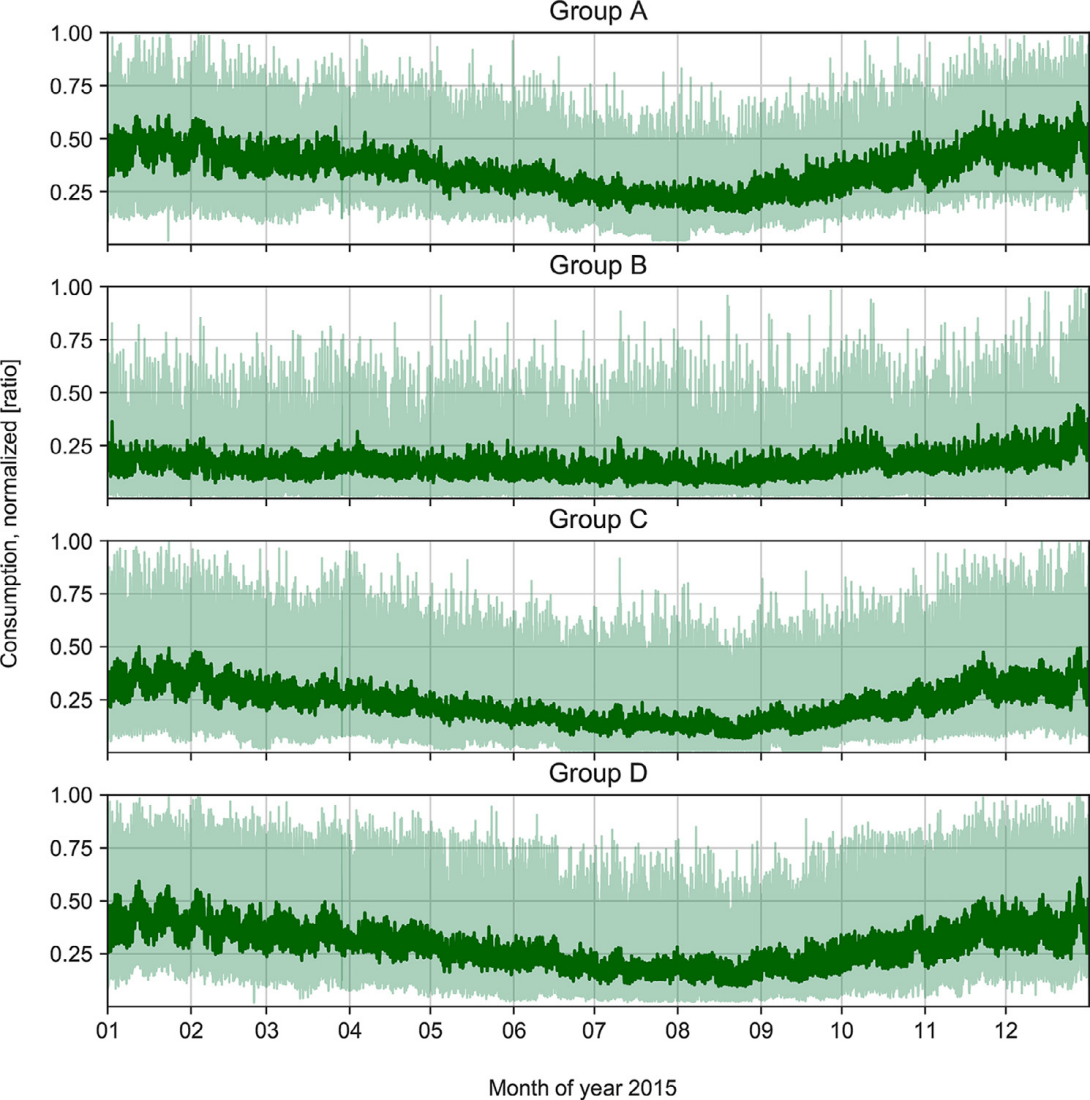
Mean (dark green) and 0.99 quantiles (light green) of clustered and normalized household consumption data for 2015 as provided by sheet *Households_raw*. (For interpretation of the references to colour in this figure legend, the reader is referred to the web version of this article.)
  It has to be noted here that in Norway heating is conducted most commonly via electrical space heaters [5], thus this series also includes the effects of such.
• *Households_filtered* shows the filtered values of the corresponding dataset as shown in Table 2 and described in the introduction of this section. The data is fitted to a normal distribution with the following parameters: mu, sigma, minimum and maximum. An excerpt of this is shown in Table 4.

The data for household consumption are given by the sheets *Households_raw* and *Households_filtered*:

• *Households_raw* consists of the load of four different household groups (Groups A, B, C and D) with hourly resolution for the time period 1 January 2015 to 31 December 2015. The (electrical) load is presented as a ratio of the maximum load over the year, and can therefore be scaled up by multiplying with the hourly peak power over the year for a given household. The four groups have been obtained by clustering smart meter data from 100 households. See Fig. 3 for a visualization of the mean and 0.99 quantiles of the household consumption.

**Table 4:** Excerpt of filtered values for group A, corresponds to sheet *Households_filtered*.

Season	Day of week	Time of day	mu	sigma	maximum	minimum
spring	weekday	hour 00–04	0.27	2.23	0.88	0.01
		hour 04–08	0.27	2.11	0.9	0.01
		hour 08–12	0.3	1.95	0.95	0.01
		hour 12–16	0.28	2.02	1	0.01
		hour 16–20	0.28	1.96	0.99	0.01
		hour 20–24	0.3	1.98	0.98	0.01

	weekend	hour 00–04	0.28	2.15	0.83	0.01
		hour 04–08	0.25	2.08	0.89	0.02
		hour 08–12	0.29	1.89	0.92	0.03
		hour 12–16	0.28	1.93	0.95	0.01
		hour 16–20	0.3	1.88	0.95	0.01
hour 20–24		0.31	1.96	0.95	0.01

### 1.2 Appliance consumption

The data for the household appliances is given by the sheet *Appliances_filtered*. It consists of filtered values of electrical loads for three different appliances: dishwasher, dryer and washing machine. The reason for the selection of these specific appliances is that these allow for load shifting, i.e. utilizing delay in their operation in order to minimize electrical consumption during peak hours. This is a common operational problem that energy community implementations could encounter and attempt to solve. The number of households for each appliance is shown in Table 5, along with information on zero values (periods when the appliance is turned off). The data is fitted to an exponential distribution with the following parameters: lambda, minimum and maximum.

**Table 5:** Appliance raw data set information.

Appliance	No. of households	Zero values (off-time)
dryer	12	79.65%
dishwasher	23	84.32%
washing machine	18	77.97%

Similar to the household consumption, the appliance consumption is provided as a ratio of the maximum load over the year, which allows for scaling by multiplying with the hourly peak power over the year. The filtered values correspond to the two-dimensional segmentation as explained in the introduction of this Section 1.

### 1.3 Electric vehicle charging

The data for electric vehicle charging is provided by the sheet *EVcharging_filtered*. It shows the filtered values for charging start probability (%) and charging duration (h). The filtered values again correspond to the segments in Table 2 and the filtering process described in the introduction of this section. The data is fitted to an exponential distribution with the following parameters: lambda, minimum and maximum. The geographical location of the data set can be observed in Fig. 2.

### 1.4 Photovoltaic power generation

**Fig. 4:**
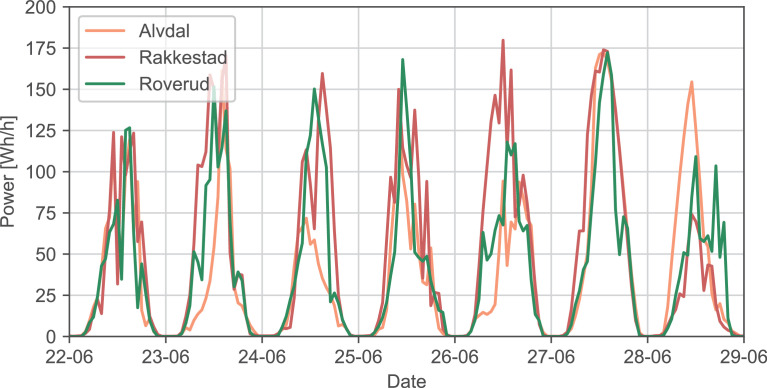
Photovoltaic power for NO1, as given in sheet *PV_raw*.

**Fig. 5:**
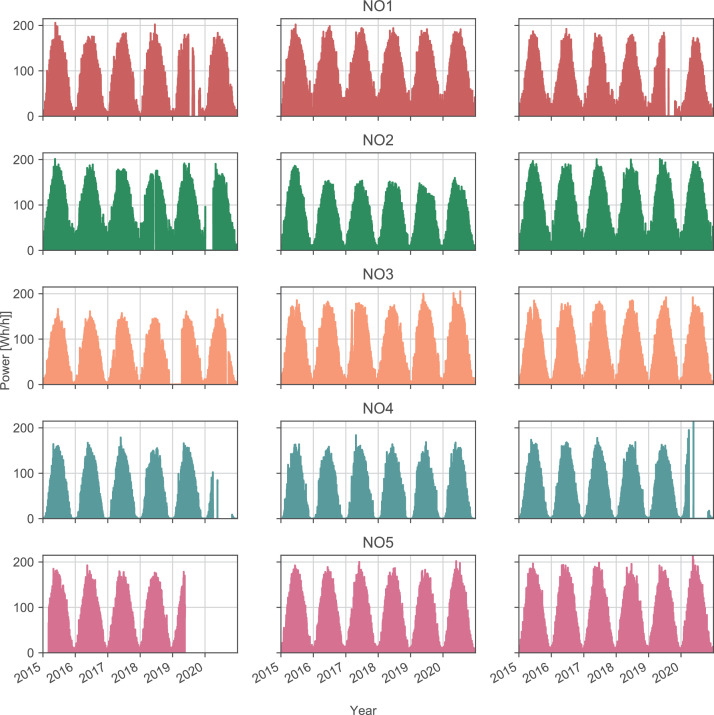
Photovoltaic power for all price areas, as given in sheet *PV_raw*.
• *PV_filtered* shows the filtered values for photovoltaic power per price area (NO1-NO5), using PV_raw as input. Again, the filtration segments are shown in Table 2 and the filtering process is described in the introduction of this section. The data is fitted to a normal distribution with the following parameters: mu, sigma, minimum and maximum.

The data for the photovoltaic power generation is presented in sheets *PV_raw* and *PV_filtered*:

• *PV_raw* consists of simulations of generated power from a photovoltaic panel of one module for 15 different locations in Norway (three selected sites located in each price area). The data is hourly for the time period 1 January 2015 to 31 December 2020. The weather stations and corresponding areas are given in Table 6, along with the data points for measured irradiance and temperature data. These irradiance and temperature data sets were used to calculate the simulated photovoltaic power as described in Section 2. Fig. 4 shows the simulated power for one week for NO1. The simulated power for each location can be seen in Fig. 5. The geographic locations of the weather stations can be observed in Fig. 2.

**Table 6:** Photovoltaic data - weather stations.

Area	Weather station	Data points irradiation	Data points temperature
NO1	Alvdal	52,097	52,378
	Rakkestad	52,599	52,604
	Roverud	49,752	50,292
NO2	Gjerpen	50,965	50,724
	Hjelmeland	52,264	52,278
	Lyngdal	52,595	52,604
NO3	Mære	52,076	52,081
	Skjetlein	50,824	50,833
	Tingvoll	52,567	52,575
NO4	Pasvik	52,602	52,602
	Tjøtta	52,229	52,231
	Holt	52,542	47,430
NO5	Kvam	38,227	36,799
	Njøs	52,551	52,553
	Ullensvang	52,590	52,594

### 1.5 Wholesale electricity price

The wholesale electricity price data is presented in sheet *WholesalePrice_filtered*. This is day-ahead market data obtained from the Norwegian electricity market Elspot operated by Nordpool and provided via a publicly accessible data platform [6]. The sheet shows filtered values for the different price zones (NO1-NO5) for the duration between 26 May 2017 to 2 April 2021. The filtration process is again conducted as described in Table 2 and in the introduction of this section. The data is fitted to a log-normal distribution with the following parameters: mu, sigma, minimum and maximum. The geographical distribution of the data set can be observed in Fig. 2.

## 2 Experimental Design, Materials and Methods

The data set allows for the formulation of control problems on the residential level. Instead of choosing specific numerical values in e.g. kWh, it was instead chosen to formulate the data in form of ratios representing usage patterns. This allows adjusting the individual data sets to various sizes of households as well as different brands and models of appliances.

As described, this data set consists of both raw and filtered data. Further, and as previously described, the filtered data was obtained by fitting the raw data to three different distributions: normal, exponential or log-normal. This was done in order to allow for utilization in stochastic models. Selection of the distribution parameters was conducted via minimization of the Kullback-Leibler divergence, whereas the utilized Python script can be found in [13]. The selection of the distribution for each specific data set was as shown in Table 7.

**Table 7:** Distributions for filtered data.

Data	Distribution	Parameters
Household consumption	Normal	mu, sigma, minimum, maximum
Appliance consumption	Exponential	lambda, minimum, maximum
Electric vehicle charging	Exponential	lambda, minimum, maximum
Photovoltaic power generation	Normal	mu, sigma, minimum, maximum
Wholesale electricity price	Log-normal	mu, sigma, minimum, maximum

In order to be fitted to the distributions shown in Table 7, the data set was normalized by feature-scaling [7], i.e.:

(1a) $$x^{\prime }=\frac{x-\text{minimum}}{\text{maximum}-\text{minimum}}$$

x is the original value

$x^{\prime }$ is the scaled value

Since the filtered data sets and their parameters are normalized, samples must be re-scaled before they can be used:

(2a) $$\begin{array}{cc} x^{\text{sample}}∼N\left(\text{mu},\text{sigma}\right) & \text{if}\;\text{Household,}\;\text{Photovoltaics}\\ x^{\text{sample}}∼\exp\left(\text{lambda}\right) & \text{if}\;\text{Appliance,}\;\text{Electric}\;\text{vehicle}\\ x^{\text{sample}}∼\mathrm{logn}\mathrm{ormal}\left(\text{mu},\text{sigma}\right) & \text{if}\;\text{Wholesale}\;\text{price} \end{array}$$

(2b) $$\mathrm{if}\;x^{\text{sample}}<0\;\mathrm{or}\;x^{\text{sample}}>1\text{:}\;\text{discard}\;x^{\text{sample}}\;\text{and}\;\text{repeat}\;\left(2a\right)$$

(2c) $$x^{\text{sample,}\;\text{re-scaled}}=x^{\text{sample}}\left(\text{maximum}-\text{minimum}\right)+\text{minimum}$$

$x^{\text{sample}}$ is the sample (with normalized values)

$x^{\text{sample,}\;\text{re-scaled}}$ is the sample scaled to real values

**Fig. 6:**
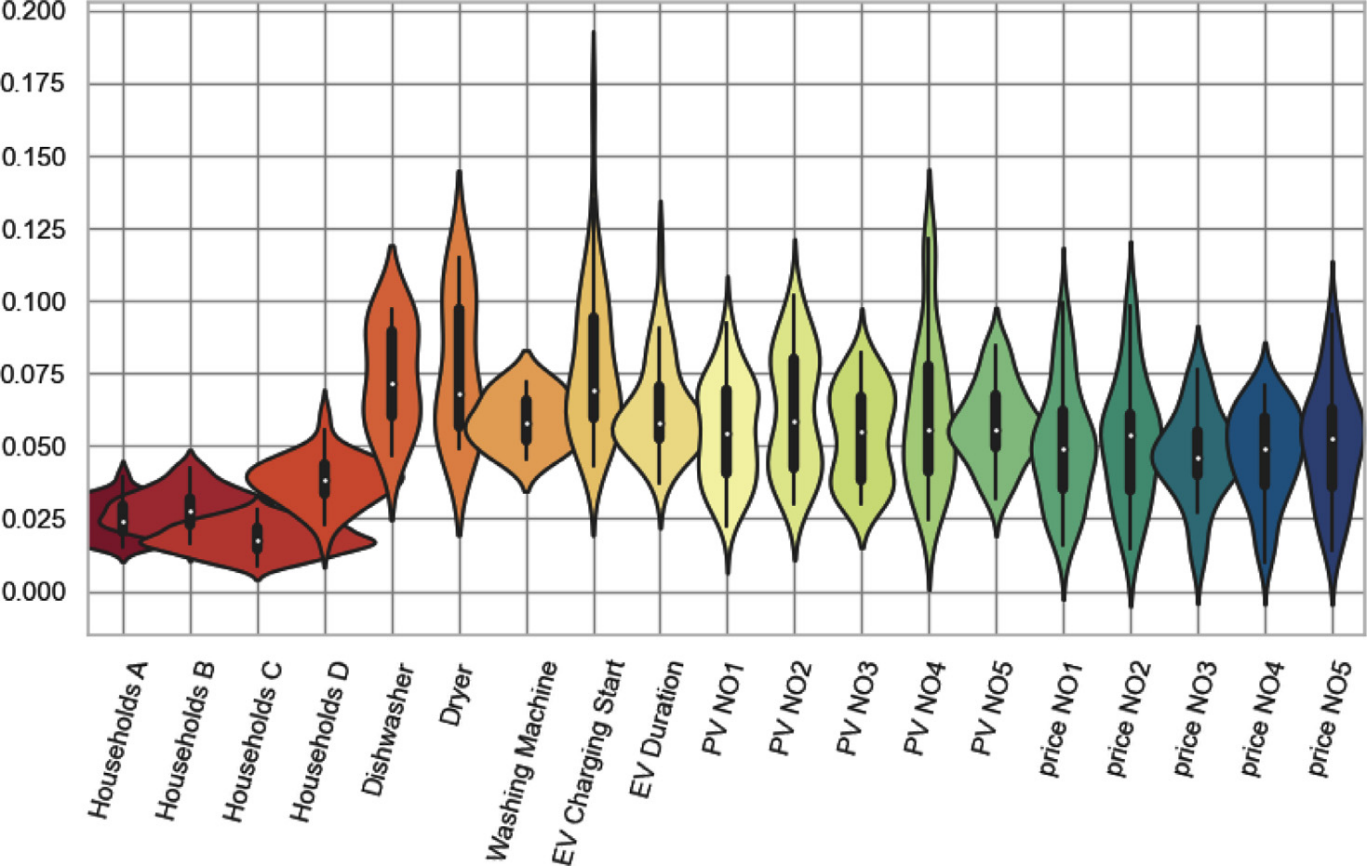
Wasserstein metric for chosen distributions..

The distributions were chosen based on the lowest Wasserstein metric for all segments. An overview of these is shown in Fig. 6.

### 2.1 Household consumption

The original household consumption data series obtained from the smart meters comes in the form of hourly resolution for year 2015. Due to data security concerns, the presented raw data was obfuscated in the following ways:

• individual household labels were removed
• the data was normalized
• instead of single households the households were aggregated into groups via k-means clustering [7]. This data set is provided in sheet *Households_raw* and illustrated in Fig. 3.

**Fig. 7:**
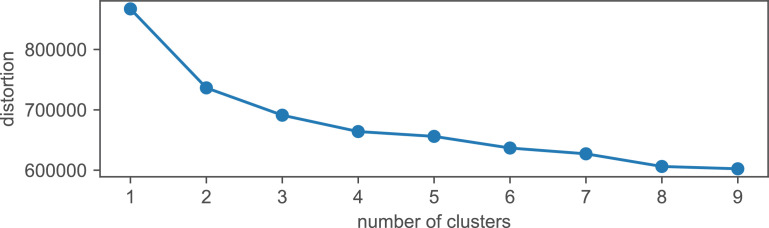
Elbow plot - household data.

The elbow plot for the segmentation obtained via clustering of the provided data series is shown in Fig. 7. Based on this, four household consumption profiles (Group A to D) were created. These groups were filtered as described above.

### 2.2 Appliance consumption

The original household appliance data series was obtained from the Norwegian research project ElDeK (Electricity Demand Knowledge, 2009–2013), in which 1-minute resolution consumption measurements in kWh of cloth washing machines, dishwashers and dryers were collected using dedicated plugin instruments [1]. These appliances all allow for load shift, i.e. postponing electricity demand to a later hour. 75 Norwegian households from four DSOs participated in the study, for periods of four weeks. The number of households for each appliance is shown in Table 5.

The data was created in the following ways:

• The 1-minute resolution measurements of dryer, washing machine and dishwasher were changed to hourly resolution by summation.
• The hourly values were normalized.
• The normalized values were filtered with a two-dimensional categorization, as explained in the introduction of Section 1.

### 2.3 Electrical vehicle charging

The electric vehicle charging data consists of charging start probability in % and charging duration in hours. The original data used to derive these series was obtained from a previously published data set [2]. More specifically, the following set was used: Dataset 1_EV charging reports.csv [8] to obtain the following information: session ID, user ID, user type, date/hour for plugin of vehicle, and date/hour for plugout of vehicle. Only data for user type = Private was used, i.e. only data for private parking spaces with one user. The data set thus consists of a number of 56 vehicles.

It has to be noted that in this data set, the number of vehicles increases over the duration of the study collecting the data points, thus leading to the following equation for the filtered data on the vehicle charging start probability:

(3) $$\text{charging}\;\text{start}\;\text{probability}\;\text{[\%]}=\frac{\text{number}\;\text{of}\;\text{vehicles}\;\text{charging}\;\text{[vehicles/h]}}{\text{total}\;\text{available}\;\text{vehicles}\;\text{[vehicles/h]}}$$

For a specific hour, the length of a charging session is then obtained via averaging the length of the charging session of all vehicles starting to charge in this hour:

(4) chargingduration[h]=mean(chargingdurationsofallvehiclesincurrenthour[h])

Finally, the data was filtered with the three-dimensional categorization, as explained in the introduction of Section 1.

### 2.4 Photovoltaic power generation

The photovoltaic data was simulated based on publicly available temperature and irradiance data. Both temperature and irradiance data in hourly intervals were obtained from [9] for the 15 weather stations as shown in Table 6 for the time period 1 January 2015 to 31 December 2020. The data was additionally cleaned for measurement errors by removing temperatures above 40 ${}^{\circ }$ C and irradiances above 1000 $W/m^{2}$. The utilized Python script can be found in [14].

Based on these time series, the resulting photovoltaic power $P_{t}$ of a single module was calculated as described in [10] of the type *Mitsubishi 255 Wp* [11]:

(5) $$P_{t}=\frac{FF\cdot I_{sc}\cdot V_{oc}\cdot T_{0}}{E_{0}\cdot \log\left(10^{6}\cdot E_{0}\right)}\cdot \frac{E_{t}\cdot \log\left(10^{6}\cdot E_{t}\right)}{T_{cell,t}}\cdot \eta_{inv}$$

where

(6) $$FF=\frac{P_{mpp}}{V_{oc}\cdot I_{sc}}$$

The cell temperature is calculated as described in [12]:

(7) $$T_{cell,t}=T_{t}+\frac{NOCT-20}{800}\cdot E_{t}+275.15$$

$P_{mpp}$ is the maximum power point of the module in W

FF is the fill factor of the module

$V_{oc}$ is the open circuit voltage of the module in V

$I_{sc}$ is the short circuit current of the module in A

$T_{cell,t}$ is the cell temperature of the module in K

$T_{t}$ is the measured temperature in degree C

$T_{0}$ is the standard module temperature in K

$E_{0}$ is the standard irradiance in $W/m^{2}$

$E_{t}$ is the measured irradiance in $W/m^{2}$

$\eta_{inv}$ is the inverter efficiency

NOCT is the nominal operating cell temperature

The resulting data is $P_{t}$ for each weather station. In addition, the values were filtered with the three-dimensional categorization, as explained in the introduction of Section 1.

### 2.5 Wholesale electricity price

The wholesale electricity price data is the spot market data obtained from the Norwegian electricity market operators’ platform [6]. The raw data obtained from the platform was filtered with the three-dimensional categorization as described previously.

## Ethics Statement

The household consumption data was collected by a DSO in Trøndelag county, Norway. The individual labels for each household was removed by the DSO. The data was further normalized (dividing by maximum value), making it impossible to identify individual households based on their maximum consumption. The specific geographic location of the 99 households is not disclosed outside of them stemming from Trøndelag county (Trøndelag county has 468,702 inhabitants).

## CRediT authorship contribution statement

**Kjersti Berg:** Software, Data curation, Writing – original draft, Writing – review & editing, Visualization. **Markus Löschenbrand:** Software, Formal analysis, Data curation, Writing – original draft, Writing – review & editing, Visualization.

## Declaration of Competing Interest

The authors declare that they have no known competing financial interests or personal relationships which have, or could be perceived to have, influenced the work reported in this article.
